# Systematic evaluation of normalization approaches in tandem mass tag and label-free protein quantification data using PRONE

**DOI:** 10.1093/bib/bbaf201

**Published:** 2025-05-08

**Authors:** Lis Arend, Klaudia Adamowicz, Johannes R Schmidt, Yuliya Burankova, Olga Zolotareva, Olga Tsoy, Josch K Pauling, Stefan Kalkhof, Jan Baumbach, Markus List, Tanja Laske

**Affiliations:** Data Science in Systems Biology, TUM School of Life Sciences, Technical University of Munich, Maximus-von-Imhof Forum 3, 85354 Freising, Germany; Institute for Computational Systems Biology, University of Hamburg, Albert-Einstein-Ring 8-10, 22761 Hamburg, Germany; Institute for Computational Systems Biology, University of Hamburg, Albert-Einstein-Ring 8-10, 22761 Hamburg, Germany; Department of Preclinical Development and Validation, Fraunhofer Institute for Cell Therapy and Immunology IZI, Perlickstr. 1, 04103 Leipzig, Germany; Institute for Computational Systems Biology, University of Hamburg, Albert-Einstein-Ring 8-10, 22761 Hamburg, Germany; Chair of Proteomics and Bioanalytics, TUM School of Life Sciences, Technical University of Munich, Emil-Erlenmeyer-Forum 5, 85354 Freising, Germany; Data Science in Systems Biology, TUM School of Life Sciences, Technical University of Munich, Maximus-von-Imhof Forum 3, 85354 Freising, Germany; Institute for Computational Systems Biology, University of Hamburg, Albert-Einstein-Ring 8-10, 22761 Hamburg, Germany; Institute for Computational Systems Biology, University of Hamburg, Albert-Einstein-Ring 8-10, 22761 Hamburg, Germany; Department of Computer Science, Vrije Universiteit Amsterdam, De Boelelaan 1111, 1081 HV, Amsterdam, The Netherlands; LipiTUM, TUM School of Life Sciences, Technical University of Munich, Maximus-von-Imhof Forum 3, 85354 Freising, Germany; Institute for Clinical Chemistry and Laboratory Medicine, University Hospital and Faculty of Medicine Carl Gustav Carus of the Dresden University of Technology, Fetscherstr. 74, 01307 Dresden, Germany; Department of Preclinical Development and Validation, Fraunhofer Institute for Cell Therapy and Immunology IZI, Perlickstr. 1, 04103 Leipzig, Germany; Fraunhofer Cluster of Excellence Immune-Mediated Diseases CIMD, Perlickstr. 1, 04103 Leipzig, Germany; Institute for Bioanalysis, University of Applied Science Coburg, Friedrich-Streib-Str. 2, 96450 Coburg, Germany; Institute for Computational Systems Biology, University of Hamburg, Albert-Einstein-Ring 8-10, 22761 Hamburg, Germany; Department of Mathematics and Computer Science, University of Southern Denmark, Campusvej 55, 5230 Odense, Denmark; Data Science in Systems Biology, TUM School of Life Sciences, Technical University of Munich, Maximus-von-Imhof Forum 3, 85354 Freising, Germany; Munich Data Science Institute (MDSI), Technical University of Munich, Walther-von-Dyck-Straße 10, 85748 Garching, Germany; Institute for Computational Systems Biology, University of Hamburg, Albert-Einstein-Ring 8-10, 22761 Hamburg, Germany; Viral Systems Modeling, Leibniz Institute of Virology, Martinistr. 52, 20251 Hamburg, Germany

**Keywords:** proteomics, label-free, isotope labeling, normalization, intragroup variation, differential expression

## Abstract

Despite the significant progress in accuracy and reliability in mass spectrometry technology, as well as the development of strategies based on isotopic labeling or internal standards in recent decades, systematic biases originating from non-biological factors remain a significant challenge in data analysis. In addition, the wide range of available normalization methods renders the choice of a suitable normalization method challenging. We systematically evaluated 17 normalization and 2 batch effect correction methods, originally developed for preprocessing DNA microarray data but widely applied in proteomics, on 6 publicly available spike-in and 3 label-free and tandem mass tag datasets. Opposed to state-of-the-art normalization practice, we found that a reduction in intragroup variation is not directly related to the effectiveness of the normalization methods. Furthermore, our results demonstrated that the methods RobNorm and Normics, specifically developed for proteomics data, in line with LoessF performed consistently well across the spike-in datasets, while EigenMS exhibited a high false-positive rate. Finally, based on experimental data, we show that normalization substantially impacts downstream analyses, and the impact is highly dataset-specific, emphasizing the importance of use-case-specific evaluations for novel proteomics datasets. For this, we developed the PROteomics Normalization Evaluator (PRONE), a unifying R package enabling comparative evaluation of normalization methods, including their impact on downstream analyses, while offering considerable flexibility, acknowledging the lack of universally accepted standards. PRONE is available on Bioconductor with a web application accessible at https://exbio.wzw.tum.de/prone/.

## Introduction

**Figure 1:**
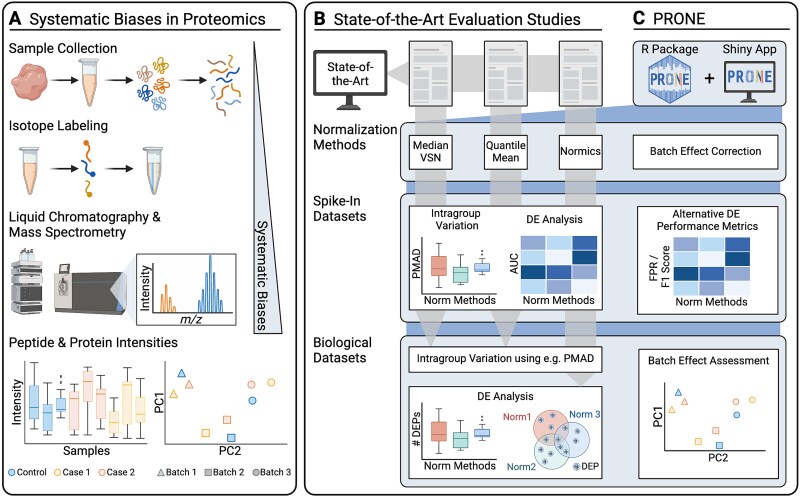
Overview of the evaluation study. (A) MS-based proteomics datasets are affected by systematic biases, attributed to variations in experiment conditions during MS analysis, ranging from sample handling to differences introduced by the instrumentation. These biases distort sample distributions and introduce potential batch effects, particularly in TMT datasets. Existing evaluation studies on proteomics normalization focus on distinct sets of normalization methods, assess intragroup variation on spike-in datasets, and predominantly use the area under the receiver operating characteristic curve (AUC) to analyze the differential expression (DE) results for each normalization method. Most studies conclude their evaluation on biological datasets after calculating intragroup variation metrics due to the absence of a known ground truth. Only very few studies proceed with examining DE results on biological datasets. To perform our comprehensive benchmarking, we developed PRONE, which integrates a broad array of normalization methods and provides batch effect correction, PRONE also expands on previous studies by calculating alternative performance metrics, including false-positive rate and F1 score, rather than relying solely on AUC values. Additionally, batch effects can be evaluated using multiple techniques within the PRONE framework.

High-throughput omics technologies nowadays produce massive amounts of data and steadily progress in detection accuracy and data generation speed [1]. However, non-biological factors arising from variations in biological experiments, sample preparation, instrumental analyses, and raw data processing frequently introduce systematic biases during an experiment (Fig. 1A) [2, 3]. Failure to address these biases can lead to erroneous results and misleading conclusions in downstream analyses, such as differential expression (DE) and functional enrichment analysis [2, 4, 5]. Given that achieving perfect experimental precision in the laboratory is nearly impossible, the critical step in compensating for this experimental variability is data normalization [1, 3]. Data normalization aims to remove or minimize these systematic biases while preserving the biological signal of interest. However, the nature and magnitude of the bias in the data is typically unknown beforehand since it is not easy to measure nor to quantify, thus the choice of a suitable approach among the wide range of available normalization methods (NMs) poses a notable challenge [2, 3, 6].

The majority of existing evaluation studies [2, 4–6] and graphical interfaces, such as Normalyzer [1], NormalyzerDE [7], and proteiNorm [8], have been developed to systematically evaluate NMs, such as median centering, quantile, linear and local regression normalization, and variance stabilization normalization (VSN) for label-free quantification (LFQ) (Fig. 1B). These studies often evaluate a diverse set of NMs on protein-level proteomics data, with many NMs originating from the DNA microarray analysis techniques. Newer approaches such as RobNorm [9] and Normics [10], specifically designed for proteomics applications, have yet to be subjected to independent validation. Importantly, most evaluations are limited in assessing intragroup variation on experimental biological datasets without known ground truth and do not extend to evaluating the impact of normalization on downstream analyses, such as DE analysis, due to the lack of a known ground truth [1, 2, 4, 7, 8]. Nevertheless, the impact of normalization on the identification and quantity of differentially abundant proteins between sample groups can be analyzed and was observed in a limited number of studies [6, 9, 10], yet this capability has not been incorporated as a feature in any tool. Furthermore, most studies restrict the evaluation on LFQ datasets. However, tandem mass tag (TMT), a chemical labeling method that enables the simultaneous mass spectrometry (MS) analysis of up to 18 samples pooled together, has become state-of-the-art in large-scale proteomic studies [11, 12]. The integration of multiple TMT batches within a single analysis introduces batch effects and thus reduces data quality. While optimized experimental design set-ups are considered to minimize batch effects [13, 14], further computational methods for batch effect correction, such as internal reference scaling (IRS) [15], are required.

While the existing studies and graphical interfaces show that a comprehensive comparative evaluation is indispensable in identifying an appropriate normalization strategy for a specific dataset, their limitations, such as the exclusion of downstream analysis evaluation and the lack of focus on TMT datasets, remain apparent. Building upon these limitations, our study aims to address the gaps by providing a systematic evaluation of a comprehensive literature-based collection of 17 NMs and two batch effect correction methods commonly employed in proteomics (Fig. 1C, see Methods). We utilized six biological proteomics datasets with known ground truth, referred to as spike-in datasets, allowing for ranking the methods in their ability to detect expected differences between sample groups. Additionally, we applied the NMs to one LFQ and two TMT biological proteomics datasets, which are absent of spike-in proteins and are referred to as biological datasets, to compare their performance in a typical real research scenario without known ground truth.

To perform this comprehensive benchmarking and streamline the selection process of optimal NMs for other proteomics data, we developed the Proteomics Normalization Evaluator (PRONE), an R package available on Bioconductor with an accompanying graphical interface accessible at https://exbio.wzw.tum.de/prone/ (Fig. 1C). PRONE incorporates 17 NMs and two batch effect correction methods in line with multiple quantitative and qualitative functions to evaluate the performance of the methods. In addition, PRONE offers an integrated solution for the complete analysis of an LFQ or TMT proteomics dataset, including missing value handling, outlier detection, and DE analysis. Unlike other interfaces that allow DE analysis based on a single selected NM, PRONE enables the comparative evaluation of DE results across multiple NMs, highlighting the impact of normalization on downstream analyses. It thus provides a robust and reliable framework for proteomics data analysis without the need to install or integrate multiple separate packages.

## Materials and methods

### Data description

#### Spike-in datasets

The experimental design of spike-in datasets, which involves adding specific proteins at known concentrations, referred to as spike-in proteins, to a constant background proteome, such as yeast lysates or human cell extracts, allows for evaluating the NMs on their ability to detect DE and calculating performance metrics. In the following, we consistently refer to proteins of the background proteome with constant concentration as background proteins. We obtained six publicly available spike-in datasets (Table 1 and Supplementary Methods): two protein standard (UPS1) spike-in (dS1–dS2, [16,17]), three *Escherichia coli* spike-in (dS3–dS5, [18–20]), and one yeast spike-in dataset (dS6, [21]).

**Table 1:** Spike-in and biological datasets. Spike-in datasets are denoted with the prefix “dS,” while biological datasets without a known ground truth are consistently denoted with “dB.” In the column for quantification type, parentheses indicate the number of TMT-batches used for the specific TMT dataset. The identifiers from ProteomeXchange.org are added for the raw data column, except for dB2, where the PDC study identifier is utilized. Due to the inconsistency in the availability of raw data from the original studies, quantification data was sourced from various other studies that have released the protein intensity matrix. The number of proteins, spike-in, and missing value rates were calculated after preprocessing the datasets (Supplementary Table 1). Further descriptions, including the utilization of instrumentation, are provided in the Supplementary Methods. Spike-in rates are not applicable (n/a) for biological datasets, since these do not contain any spike-in proteins.

**Dataset**	**Type**	**Quantification type**	**Raw data (ID)**	**Quantification data**	**Number of proteins**	**Spike-in rate (%)**	**MV rate (%)**
dS1	UPS1 spike-in (4 levels)	LFQ	Tabb *et al.* [16] (CPTAC Study 6)	Välikangas *et al.* [2]	731	4.92	0.31
dS2	UPS1 spike-in (6 levels)	LFQ	Ramus *et al.* [17] (PXD001819)	Graw *et al.* [8]	656	3.05	3.2
dS3	*E.coli* spike-in (5 levels)	LFQ	Shen *et al.* [18] (PXD003881)	Sticker *et al.* [22]	4112	14.64	1.36
dS4	*E.coli* spike-in (2 levels)	LFQ	Cox *et al.* [19] (PXD00279)	Cox *et al.* [19] (PXD00279)	4951	30.9	1.4
dS5	*E.coli* spike-in (3 levels)	TMT 10-plex (1)	Zhu *et al.* [20] (PXD013277)	Phil Wilmarth [23]	9650	21.67	0
dS6	Yeast spike-in (3 levels)	TMT 11-plex (1)	O’Connell *et al.* [21] (PXD007683)	Ammar *et al.* [24]	8341	14.51	0.002
dB1	Osteogenic differentiation of hPDLSCs (4 time points)	TMT 6-plex (3)	Li *et al.* [25] (PXD020908)	MaxQuant executed in-house	4848	n/a	0.003
dB2	Ovarian carcinoma JHU	TMT 10-plex (13)	Hu *et al.* [26] (PDC000110)	MaxQuant executed in-house	7207	n/a	1.91
dB3	AROM+ transgenic versus wild-type mice	LFQ	Vehmas *et al.* [27] (PXD002025)	Vehmas *et al.* [27] (PXD002025)	1450	n/a	0.06

#### Biological datasets

To demonstrate the practical application of PRONE on biologically meaningful data, we obtained one publicly available label-free and two TMT datasets. The first dataset, dB1, from a cell culture study of Li *et al.* [25] focuses on proteome dynamics during osteogenic differentiation of human periodontal ligament stem cells (hPDLSCs). The second, dB2, is a clinical dataset from the ovarian carcinoma Johns Hopkins University proteome study of Hu *et al.* [26]. Lastly, dB3, an LFQ dataset of Vehmas *et al.* [27], explores the effects of high estrogen-to-androgen ratio on the mice liver proteome (Table 1 and Supplementary Methods).

### Review and selection of normalization methods for proteomics data

A selection of 17 normalization and two batch effect correction methods was made based on a comprehensive literature review (Table 2 and Supplementary Methods). Previous works [1, 2, 4, 6–8, 28] gave a systematic review of commonly used NMs in proteomics data. Notably, many of the methods applied on proteomics data have originally been developed for DNA microarray analyses. In contrast, the novel approaches RobNorm [9] and two variants of Normics [10] were included as the approaches have only been assessed in their original publication and were specifically developed for proteomics data. Phil Wilmarth, a data analyst focusing on the analysis of TMT experiments, provided a detailed evaluation of three NMs, GlobalMean, IRS, and TMM, available online [29].

**Table 2:** Summary of the normalization methods and batch-effect correction methods. We summarized the selected approaches into five categories, based on the nomenclature proposed by Wang *et al.* [9]. The column scale specifies if normalization was applied on raw or log2-transformed data, based on recommendations found in the literature for each respective normalization method. Of note, this is one of several parameters that can be easily configured in PRONE. More detailed descriptions of the normalization approaches and batch effect correction techniques are provided in the Supplementary Methods.

**Category**	**Approach**	**Abbreviation**	**Origin**	**Scale**	**Previous evaluation studies**
Simple sample shifting	Mean and median normalization	Mean, Median	Microarray	Raw [7]	[1,2,6–10,28,30]
	Global intensity normalization	GlobalMean, GlobalMedian	Microarray	Raw [7,29]	[1,7,8,15,29]
	Median absolute deviation normalization	MAD	Proteomics	log2 [7]	[1, 7]
	Normics with median normalization	NormicsMedian	Proteomics	Raw [10]	[10]
Sample-to-reference	Quantile normalization	Quantile	Microarray	log2 [7]	[1,2,4,6–8,10,28,30,31]
	Linear regression normalization	Rlr, RlrMA, RlrMACyc	Microarray	log2 [7]	[1, 2, 4, 7, 8, 28]
	Local regression normalization	LoessF, LoessCyc	Microarray	log2 [7]	[2,4,7–10,28,30]
	Trimmed mean of M-values normalization	TMM	RNA-seq	Raw [29,32]	[23,29]
Variance stabilizing	Variance stabilizing normalization	VSN	Microarray	Raw [7,33]	[1,2,7–10,28,31]
	Normics with VSN	NormicsVSN	Proteomics	Raw [10]	[10]
Model-based	EigenMS	EigenMS	Metabolomics	log2 [34]	[2, 5, 9]
	RobNorm	RobNorm	Proteomics	log2 [9]	[9]
Batch effect correction	Internal reference scaling	IRS	Proteomics	Raw [29]	[15,29]
	limma::removeBatchEffects	limBE	Microarray	log2 [33]	[33]

### Evaluation of the normalization methods

#### Intragroup and intrabatch variation

Intragroup variation of all sample groups was measured using the pooled median absolute deviation (PMAD) for each NM since the PMAD is less affected by outliers than the intragroup pooled variance estimate or the pooled coefficient of variation [1]. The PMAD of a sample group *g* is defined as the average median absolute deviation (MAD), calculated on the samples of group *g*, over all the proteins *i* = 1, …, *n*.


$$ {PMAD}_g=\frac{\sum_{i=1}^n\ {MAD}_{ig}}{n} $$

with *g*_1_, …, *g_e_* being the indices of the samples of condition *g*, and *e* being the number of samples of condition *g*. Additionally, the Pearson correlation coefficient was calculated for each pair of samples of a condition to determine the degree of similarity between the technical replicates in sample groups, with a high value indicating high intragroup similarity.

For the biological TMT datasets, diagnostic plots were used for visual evaluation, and a silhouette coefficient–based alternative strategy to PMAD was implemented to quantitatively evaluate the performance of normalization and batch effect correction methods. The consistency of biological (condition) and technical (TMT batch) sample groups was assessed for each NM using principal components (PCs) and the silhouette coefficient, as described in [35]. Initially, the Euclidean distance between all samples was computed based on the first three PCs, as in [36]. Then, the silhouette coefficient was used to quantify how well a sample fits within its assigned sample group (condition or TMT batch) compared to other sample groups, with values close to 1 indicating correct group assignment and values close to −1 suggesting the sample belongs to a different sample group.


$$ {S}_j=\frac{b_j-{a}_j}{\max \left\{{a}_j,{b}_j\right\}} $$

Here, ${a}_j$ denotes the average Euclidean distance over the first three PCs of sample $j$ and all other samples in the same sample group, and ${b}_j$ is the minimum average distance between sample $j$ and samples in all other sample groups [35, 36].

Finally, the average silhouette coefficient for all samples within a (condition or TMT batch) group was calculated to summarize the findings. In this context, a batch coefficient close to 1 indicates the presence of batch effects, while a condition coefficient close to 1 reflects a strong biological effect.

#### Differential expression analysis

DE of proteins was examined in each two-group comparison using limma [33] after the application of the different NMs in all spike-in and biological datasets. To ensure clarity, all comparisons are in the form of Case–Control. For instance, when the logFC is positive, the protein intensity is greater in the Case compared to the Control. Furthermore, we applied the reproducibility-optimized test statistic (ROTS) [37] on spike-in datasets, as performed by Välikangas *et al.* [2], to evaluate the impact of the DE method on the assessment of NMs. All *P*-values mentioned in this study have been adjusted per NM and dataset to control the false-discovery rate using the Benjamini–Hochberg procedure at a significance level of 0.05. In addition, a threshold of |logFC| >1 was applied for the biological datasets.

Since the ground truth is known for the spike-in datasets, performance metrics such as area under the receiver operating characteristic curve (AUC), false-positive rates (FPRs), and F1 scores were calculated. The calculation of an F1 score is particularly useful in cases with imbalanced datasets, such as in the spike-in datasets, as it integrates precision and recall into a single metric by using the harmonic mean. Since optimizing one metric can negatively influence the other, the F1 score provides a harmonized solution. In this context, a spike-in protein is counted as true positive when detected as DE while as false negative if not. In contrast, a background protein not DE is counted as true negative, while one exhibiting significant DE is classified as false positive (FP).

Finally, since biological datasets lack a known ground truth, we compared the number of DE proteins and conducted an intersection analysis on the DE results to evaluate the consistency of DE proteins obtained with the different NMs. For this purpose, we calculated the Jaccard similarity coefficient of DE proteins between all pairs of NMs, defined as the size of the intersection divided by the size of the union of the sets.


$$ J\left(A,B\right)=\frac{\left|A\cap B\right|}{\left|A\cup B\right|} $$

Here, *A* and *B* represent the sets of DE proteins identified by two NMs.

#### Functional enrichment analysis

Finally, we conducted a biological functional analysis using PRONE, which internally uses g:Profiler [38] to further examine the DE results obtained from the different NMs on the biological datasets. In this analysis, significantly enriched KEGG pathways [39] (*P*-value <0.05) of all NMs were intersected in a similar manner to the DE results, employing the Jaccard similarity coefficient.

## Results

### PRONE: a competitive, accessible, and adaptable tool challenging state-of-the-art interfaces

#### Comparative evaluation of state-of-the-art tools

Open-source tools such as NormalyzerDE [7] and proteiNorm [8] were primarily designed to systematically assess NMs. In contrast, proteoDA [40], tidyproteomics [30], and AlphaPeptStats [31] offer an extensive range of functionalities for proteomics data analysis (Table 3). All five software tools are constrained in their data preprocessing features, such as missing value filtering and outlier detection. A collection of NMs was curated from the literature (Table 2), yet none of the packages offer all the techniques (Table 1). Among the state-of-the-art tools, only AlphaPeptStats offers batch effect correction, restricting their analyses to datasets not measured in multiple batches. Although each tool integrates various DE methods, none allows the comparative assessment of NMs on DE analysis. However, this is of high importance since the choice of NM has a direct influence on all downstream analyses [6, 9, 10]. Moreover, the tools lack functionality for calculating performance metrics, such as FPRs and F1 scores, in spike-in datasets, thereby preventing the utilization of the known ground truth for evaluating NMs. As most of the studies presenting a novel NM or assessing multiple NMs include at least one spike-in dataset for evaluation, a tool offering more functionalities specific for spike-in datasets is lacking.

**Table 3:** Comparative overview of publicly available state-of-the-art tools and PRONE. NormalyzerDE and proteiNorm, primarily designed for data normalization, and the tools proteoDA, AlphaPeptStats, and tidyproteomics, developed to facilitate proteomics data analysis, were compared along with PRONE (with light blue background) on various functionalities. Notably, since proteoDA is an extension of proteiNorm, these two were combined for comparison. ^*^Filter proteins based on additional attributes, such as “Reverse” or “Potential contaminant”, as provided in MaxQuant output files. ^**^In RT-segmented normalization, data points are grouped by their retention times into distinct segments, where normalization is performed individually within each segment to account for variations in electrospray ionization intensity [7].

**Functionalities**	**NormalyzerDE**	**proteiNorm/proteoDA**	**AlphaPeptStats**	**tidyproteomics**	**PRONE**
Interface	R package + web interface	R package + web interface	Python package + web interface	R package + web interface	R package + web interface
Level	Peptide- and protein-centric	Peptide- and protein-centric	Protein-centric	Peptide- and protein-centric	Protein-centric
Filter proteins by annotation^*^	×	✓	✓ (contaminant removal)	✓	✓
Missing value report and filtering	×	✓	×	×	✓
Outlier detection	×	Only manual filter function available	×	Only filter function available based on annotations	POMA [41] and manual filter available based on visualizations
No. of normalization methods	7 + RT-segmented normalization^**^	8	4	7	17
Intragroup variation metrics	✓ (5 metrics)	✓ (5 metrics)	×	✓ (4 metrics)	✓ (5 metrics)
Evaluation plots (e.g. boxplots, PCA plots)	✓	✓	✓	✓	✓
Batch effect correction	×	×	✓	×	✓ (2 approaches)
Imputation	×	✓ (6 approaches)	✓ (4 approaches)	✓ (4 approaches)	✓ (1 approach)
DE analysis	limma, ANOVA	DAtest [42]	ANOVA, ANCOVA, *t*-test, Tukey test	*t*-test, limma	limma, DEqMS, ROTS
Performance metrics for spike-in datasets	×	×	×	×	✓
DEP count comparison	×	×	×	×	✓
Intersection analysis	×	×	×	×	✓
Functional enrichment	×	×	✓	✓	×

#### Overview of PRONE

**Figure 2:**
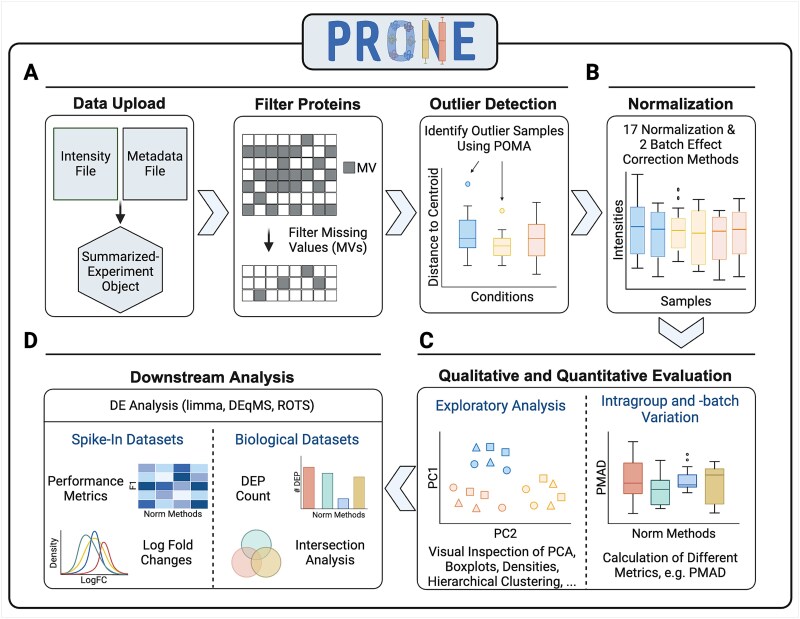
Overview of the six-step workflow of PRONE for proteomics data analysis and normalization method evaluation. This six-step process initiates with the import of LFQ or TMT proteomic and two preprocessing steps (A). It allows for the exclusion of proteins exhibiting excessive missing values and the identification of outlier samples using POMA. The workflow includes 17 normalization methods and two batch effect techniques, which can be applied simultaneously and sequentially (B). Subsequent stages involve evaluating the performance qualitatively and quantitatively through the calculation of intragroup and intrabatch variation metrics and exploratory data analysis (C). Finally, three differential expression (DE) analysis methods are integrated to assess the efficacy of the applied strategies in detecting DE proteins (D). Thanks to the known ground truth of spike-in datasets, the evaluation of DE results is based on metrics, such as F1 scores. In contrast, the evaluation of DE results of biological datasets is based on intersection analyses of DE results obtained by the various normalization methods.

PRONE is a six-step workflow to benchmark the effectiveness of multiple NMs on protein-centric LFQ or TMT spike-in and biological proteomics data and guide the user’s decision on the NM (Fig. 2). It offers the broadest selection of data normalization and batch effect correction techniques available.

As input, PRONE requires two files: the raw protein intensity table and a metadata file (Fig. 2A). PRONE is currently limited to protein-centric data, requiring the input protein intensity file to be in a tabular format, with proteins represented by rows and samples identified by columns. Other additional columns originating from MaxQuant [43], for instance, are allowed and can be accessed at any time. The metadata file needs to specify, at minimum, the sample names and their corresponding group classifications.

Since the quality of MS-based proteomics data is challenged by the presence of missing values, users can specify a threshold for the minimal percentage of samples with a valid value for a given protein. Proteins not fulfilling this criterion can be filtered out. For researchers in favor of imputation rather than protein filtering, we provide a mixed imputation approach [44]. This approach applies *k*-nearest neighbor imputation for proteins with missing values assumed to be missing at random, while values missing not at random are imputed using random draws from a left-shifted Gaussian distribution. A protein is classified as having missing values not at random if it exhibits missing values across all replicates of at least one condition. In addition, we included the multivariate outlier detection method of POMA [41]. POMA computes the centroids of sample groups and employs a threshold on the interquartile range of each sample relative to its group centroid to identify outliers specific to each sample group. POMA outlier samples can be examined using various visualization techniques and subsequently either retained or excluded from the dataset.

PRONE provides considerable flexibility to users concerning normalization acknowledging the lack of universally accepted standards (Fig. 2B, Table 2). For instance, batch effect correction can be executed either before or after normalization, and users decide whether to apply normalization on raw or log2-transformed data. The results of the NMs can then be compared using exploratory data analysis and visualizations, such as boxplots and PCA plots, or by calculating different intragroup variation metrics, e.g. pooled median absolute deviation (PMAD) (Fig. 2C). Additionally, hierarchical clustering of samples and the computation of silhouette coefficients based on PC components are provided to identify potential TMT-batch effects (see Methods) [14, 35]. Notably, PRONE offers the option to display evaluation metrics and data visualizations for a single NM at a time or to compare various NMs in a single plot.

DE analysis (Fig. 2D) can be conducted using limma [33], ROTS [37], or DEqMS [20]. Given the known ground truth in spike-in datasets, various standard metrics, including F1 scores, can be employed to assess NMs in detecting DE proteins. Furthermore, it is possible to compare the calculated logarithmic fold changes at base 2 (logFCs) against the expected logFCs derived from the known spike-in shifts. In biological data scenarios lacking a ground truth, alternative evaluation strategies are necessary. These include summarizing the counts of DE proteins across NMs and conducting intersection analyses of DE findings. While DE intersection was previously conducted by other authors [10, 28], it has not yet been incorporated into an R package or graphical user interface before PRONE. Jaccard similarities are calculated to evaluate the consistency of DE proteins identified using different NMs (see Methods). Furthermore, the DE results of the different NMs can be assessed through biological functional enrichment analysis using g:Profiler, similar to [9, 10].

Notably, PRONE provides features for preprocessing, normalization, evaluation of NMs, and downstream analysis, which extends its utility beyond normalization as an integrated platform for the comprehensive analysis of proteomics data. PRONE is available as an R Bioconductor package and a user-friendly R Shiny App, accessible under https://exbio.wzw.tum.de/prone, to provide access to the functionalities for analyzing biological proteomics data without extensive programming knowledge.

### Spike-in datasets

We downloaded six publicly available spike-in datasets (Table 1) and used PRONE to compare the NMs in their ability to reduce intragroup variation and detect DE fold-changes (see Methods and Supplementary Methods). A standardized preprocessing strategy was applied to all spike-in datasets, encompassing protein filtering based on multiple criteria, such as missing value filtering and potential contaminant removal, and outlier sample detection (Supplementary Table 1). Briefly, we highlight key differences in spike-in datasets relevant for discussion of normalization performance. Detected proteins range from 656 (dS2) to 9650 (dS5), with dS2 exhibiting the highest missing value rate (Table 1). UPS1 spike-in datasets (dS1, dS2) exhibit lower spike-in rates (<5%) and higher sample-specific variability (Supplementary Fig. 1). Specifically, spike-in and background protein distributions vary across both conditions and samples in dS1–dS3, while dS5 and dS6 show the most consistent background distributions. Each dataset was subjected to all 17 NMs (Table 2).

#### Impact of normalization on intragroup variation

**Figure 3:**
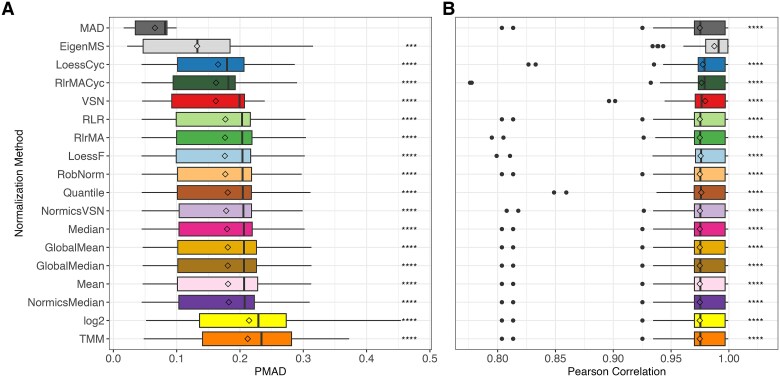
Overall intragroup variation and similarity of normalization methods in spike-in datasets. Boxplots depicting the distribution of pooled median absolute deviation (PMAD, A) and Pearson correlation coefficients (B) for all sample groups across all spike-in datasets, with normalization methods and unnormalized log2-transformation (“log2”) ranked by their median PMAD. Diamonds represent mean PMAD and Pearson correlation in (A) and (B), respectively. Paired Wilcoxon rank-sum tests were conducted to compare the PMAD and Pearson correlation values of MAD and EigenMS, respectively, with those of all other normalization techniques. Detailed results per spike-in dataset are provided in Supplementary Tables 2 and 3. ^*^P ≤ 0.05, ^**^P ≤  0.01, ^***^P ≤  0.001, ^****^P ≤  0.0001.

Similar to previous evaluation studies [1, 2, 4, 8, 9], the PMAD and Pearson correlation coefficients were computed for each sample group within a dataset and across all NMs to assess their impact on intragroup variation and sample similarity. Normalization mainly decreased intragroup variation between technical replicates in all datasets compared to unnormalized log2-transformed data, except TMM (Fig. 3A and Supplementary Table 2). MAD consistently achieved the highest percent reduction across all spike-in datasets (Supplementary Table 2).

Consistent findings were noticed across the spike-in datasets regarding the intragroup similarity between technical replicates measured using the Pearson correlation coefficient (Fig. 3B and Supplementary Table 3). EigenMS was an exception and showed a most noticeable increase in correlation. In contrast to the observed differences in intragroup variation, MAD normalized intensities did not exhibit a substantial difference in sample correlation compared to other NMs. LoessCyc, VSN, and RlrMACyc had an overall higher median of Pearson correlation over the sample groups in all spike-in datasets.

#### Effect of normalization on differential expression

**Figure 4:**
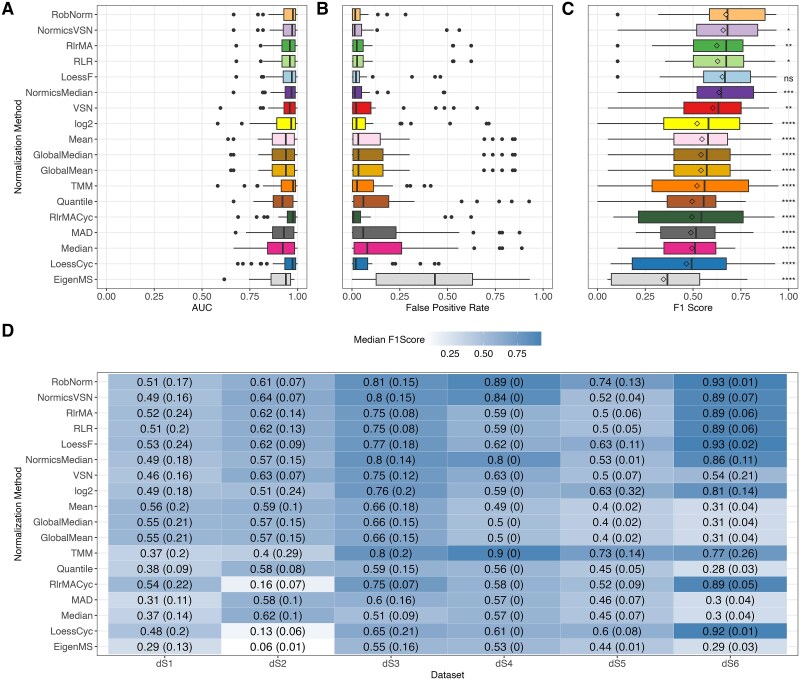
Effect of normalization on differential expression and comparison of evaluation metrics. Differential expression analysis was conducted on every pairwise comparison of each spike-in dataset using limma with a threshold of 0.05 on Benjamini–Hochberg-adjusted *P*-values. Area under the receiver operating characteristic curve (AUC) values (A), FPRs (B), and F1 scores (C) were calculated for all normalization methods across all pairwise comparisons and spike-in datasets. Diamonds in panel (C) represent mean F1 scores. Paired Wilcoxon rank-sum tests were conducted to compare the F1 scores of RobNorm with those of all other normalization techniques. Median F1 scores with the median absolute deviations indicated in brackets separated by spike-in dataset are shown in (D). The higher the F1 score, the better the performance of the normalization method. For all panels, normalization methods were sorted according to the median F1 score over all datasets. ^*^P ≤  0.05, ^**^P ≤  0.01, ^***^P ≤ 0.001, ^****^P ≤  0.0001, ns = not significant.

DE analysis was conducted for each spike-in dataset across all pairwise comparisons using limma [33] with multiple testing corrections using Benjamini–Hochberg procedure [42], setting an adjusted *P*-value threshold of 0.05. Since prior research predominantly assessed the DE results of NMs using AUC values [2, 7, 9], we calculated the AUC for every pairwise comparison and each spike-in dataset (Fig. 4A). The AUC analysis suggests high levels of performance, as indicated by median AUC values exceeding 0.75 across all NMs. However, in this study, we observed elevated FPRs in the spike-in datasets (Fig. 4B and Supplementary Fig. 2A), highlighting the limitations of relying solely on AUC values. Due to these observations and the imbalance of spike-in and background proteins (Table 1), we employed F1 scores for the conclusive assessment of the DE outcomes (Fig. 4C and4D).

The analysis revealed significant fluctuations in F1 scores across the different spike-in datasets (Fig. 4D). TMM exhibited lower performance in dS1–dS2 compared to the other datasets, while the cyclic regression NMs performed well in dS6 but showed reduced efficacy in dS2. The high F1 scores of TMM in dS3–dS6 are primarily due to low FPRs relative to other NMs (Supplementary Fig. 2A). Generally, most notable shifts in F1 scores across NMs (Fig. 4) can be attributed to background proteins being misclassified as DE (Supplementary Fig. 2A). For instance, simple sample shifting methods and quantile normalization exhibit a higher FPR in dS6 compared to more complex approaches. In dS2, the linear and local regression methods employing a reference sample, including RlrMA, RLR, and LoessF, demonstrated overall high median F1 scores (>0.6) compared to their cyclic counterparts (<0.2) (Fig. 4D), driven by a pronounced reduction in TPs (Supplementary Fig. 2B).

Overall, RobNorm performed significantly better than all other methods (Fig. 4C and D) except LoessF (Wilcoxon test *P*-value =  0.1, Supplementary Fig. 3). NormicsVSN, RlrMA, and RLR ranked among the top-performing methods based on the median F1 score across all spike-in datasets and pairwise comparisons (Fig. 4C). In contrast, with EigenMS, the median F1 score is below 0.4, which aligns with its significantly higher FPRs (Fig. 4B).

#### Effect of normalization on log fold changes

Due to the experimental design of spike-in datasets, it is anticipated that the concentrations of the spike-in proteins will exhibit variability, whereas the concentrations of the background proteins are constant. The logFCs of the spike-in proteins were typically underestimated both in the normalized data and in log2-transformed data (Supplementary Fig. 4A). In general, the tested methods yielded comparable results in estimating logFCs of the spike-in proteins, with the exception of MAD, which consistently produced lower logFCs of the spike-in proteins than expected. The density distributions of the spike-in dataset dS2 illustrate the underlying reason for this effect (Supplementary Fig. 5). Specifically, MAD normalization strongly compresses intensity values, leading to a significant reduction in overall variance and systematically lower logFC values.

While the logFC values of the background proteins should remain unchanged, the logFC distributions of the background proteins across all datasets were not centered at zero for the majority of the NMs (Supplementary Fig. 4B). Nonetheless, the distributions of the logFCs in datasets normalized using RobNorm, LoessF, and LoessCyc were notably concentrated around zero. Of these, RobNorm demonstrated the most accurate outcome (median logFC = 0.00109).

#### Impact of differential expression method on proteomics data analysis

To address the observation that limma tends to detect a high amount of FPs in spike-in datasets (Fig. 4B), we integrated another method, reproducibility-optimized test statistic (ROTS) test, into the pipeline. This integration was based on prior studies demonstrating ROTS’ enhanced performance in detecting fold-differences over the conventional *t*-test in proteomics data analysis [2, 37, 45]. While PRONE also includes DEqMS as an alternative to limma and ROTS, its performance was not evaluated in this study due to the limited availability of peptide counts per protein in the majority of the spike-in datasets.

Overall, ROTS consistently reduced the number of FPs across all NMs (Supplementary Fig. 6A). The reduction in FPs by ROTS was associated with a slight decrease in the number of TPs in various NMs, impacting the ranking of NMs based on F1 scores (Supplementary Fig. 6B). The cyclic regression methods demonstrated improved performance following the application of ROTS, and LoessF emerged as the best-performing method based on the median F1 score followed by RobNorm, NormicsVSN, NormicsMedian, and VSN, though the differences in performance were not significant (Wilcoxon test *P*-values >0.05). Despite these alterations, the methodologies that previously showed best performance according to F1 scores based on limma DE results, specifically RobNorm and NormicsVSN, continued to rank among the top-performing methods with the adoption of ROTS. Finally, the F1 scores of ROTS and limma were compared in a paired Wilcoxon rank-sum test per NM (Supplementary Fig. 6C). For certain NMs, i.e. TMM, RlrMACyc, and LoessCyc, significant differences were observed in the F1 scores between ROTS and limma, whereas for the best-performing methods, LoessF, RobNorm, and NormicsVSN, no significant differences were detected. Thus, while the choice of DE method influences the performance evaluation of the NMs, it does not substantially alter the ranking of the best-performing methods in our specific cases.

### Biological datasets

While spike-in datasets offer a known ground truth for method validation, the absence of such ground truth in biological datasets complicates the selection of an appropriate NM. To illustrate the application of PRONE in typical biological study settings, we selected one LFQ and two TMT datasets (see Methods and Table 1). Following the preprocessing of the datasets with PRONE (Supplementary Table 4), we primarily focused on evaluating intragroup variation and comparing DE results. Based on the recommendations of [10, 46], we evaluated VSN and NormicsVSN with the default lts.quantile value of 0.9, as well as with the lts.quantile parameter set to 0.5, hereafter referred to as VSN_0.5 and NormicsVSN_0.5 in this study.

#### Impact of normalization on intragroup and intrabatch variation

**Figure 5:**
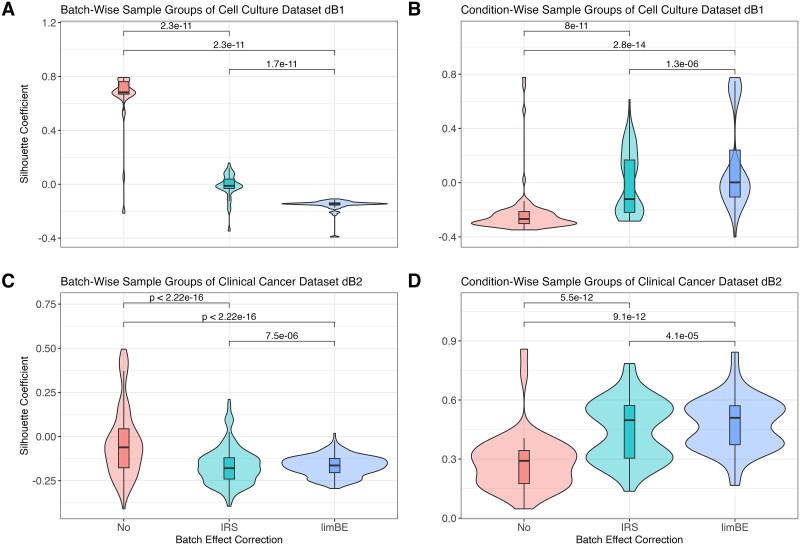
Batch effect assessment of the biological TMT datasets using silhouette coefficients per normalization method. The silhouette coefficients, based on the Euclidean distances among samples derived from the first three principal components, quantify how well each sample fits within its assigned group. The silhouette coefficients were computed for each sample group, TMT-batch- and condition-wise across all combinations of normalization techniques and batch effect correction techniques in the cell culture dataset dB1 (A, B) and the clinical cancer dataset dB2 (C, D), respectively. Normalization methods were applied without batch effect correction (No), with internal reference scaling (IRS), and with limma::removeBatchEffects (limBE). Paired Wilcoxon rank-sum tests were conducted to compare the silhouette coefficients of the batch effect correction methods.

In the cell culture dataset dB1 (Table 1), we examined the biological replicates across the four time points. Upon normalization using PRONE’s 17 NMs, hierarchical clustering of samples revealed clear evidence of TMT-batch effects according to TMT labeling for all NMs, except for EigenMS (Supplementary Fig. 7A). The application of batch effect correction methods, IRS and limBE, on normalized data mitigated this technical variability to some extent. Notably, the biological signal, corresponding to timepoints, became more pronounced with limBE for the majority of NMs (Supplementary Fig. 7B). However, since diagnostic plots rely on visual interpretation, quantitative approaches offer a more objective assessment of batch effect removal while preserving the underlying biological signal. Given the limitations of the PMAD metric in the spike-in datasets, an alternative approach based on PCs and silhouette coefficients was used (see Methods), where a coefficient of 1 denotes ideal goodness of clustering with respect to TMT batch (technical bias) or condition (biological signal), respectively. The silhouette coefficients of TMT-batch groups were significantly reduced following the application of either IRS or limBE, a trend observed across all NMs except EigenMS (Supplementary Fig. 8). For simplicity, we summarized the silhouette coefficients for all NMs with limBE showing the most substantial reduction in median silhouette coefficient overall (Fig. 5A). Notably, similar to the results observed using the hierarchical clustering, the biological signal, e.g. related to different time points, remained preserved in the data after correcting for TMT-batch effects (Fig. 5B).

This finding was further validated using the clinical cancer dataset dB2. The hierarchical clustering of samples showed that without batch effect correction, samples exclusively group by TMT batch, while batch effect correction mitigates this effect (Supplementary Fig. 9). Notably, samples normalized with EigenMS cluster by pathological status rather than TMT batch without the use of any batch effect correction method. Furthermore, as already observed in the cell culture dataset dB1, limBE efficiently removes the TMT-batch effects and makes the biological signal more prominent, which is in concordance with the silhouette coefficients (Fig. 5C, Fig. 5D, and Supplementary Fig. 10).

For completeness, the intragroup variation metric PMAD used in the spike-in datasets was computed on the biological TMT datasets as well (Supplementary Fig. 11). The results align with the silhouette coefficients (Fig. 5). Consequently, we proceeded with the downstream analysis using data from all NMs following the application of limBE. In contrast, for the LFQ dataset dB3, all NMs were applied without any batch effect correction as samples were not measured in different TMT batches.

#### Impact of normalization on differential expression results of biological proteomics data

**Figure 6:**
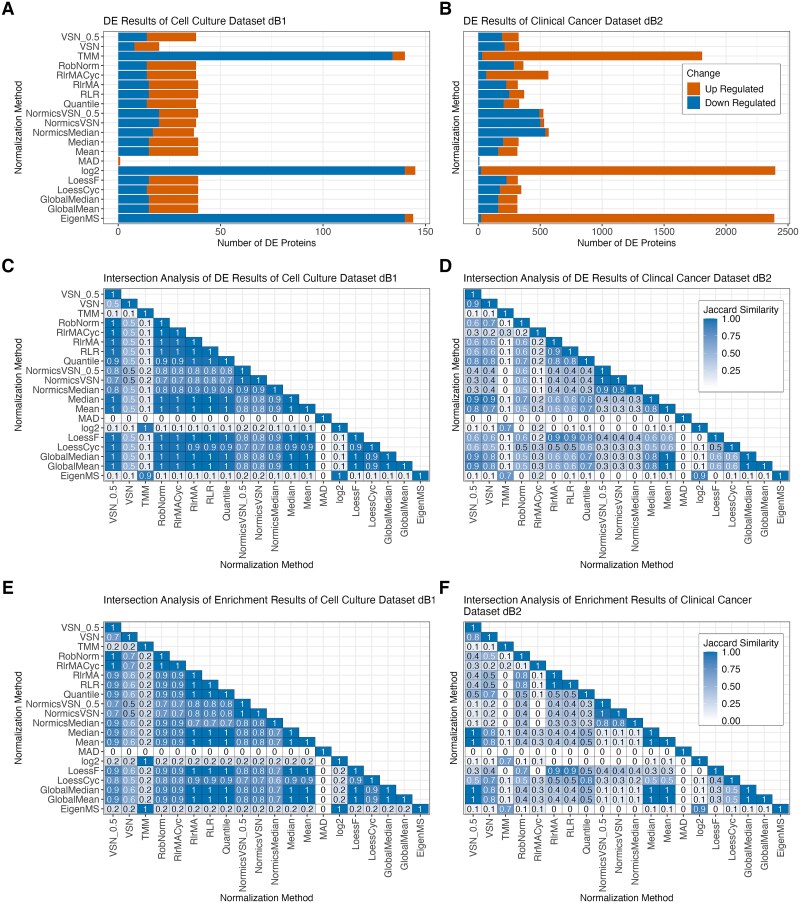
Differential expression results of biological TMT datasets. Bar plots showing the number of differentially expressed (DE) proteins of the pairwise comparison D14–D0 for each normalization method with limBE applied on top to remove TMT-batch effects in the cell culture dataset dB1 (A) and the clinical cancer dataset dB2 (B), respectively. DE proteins are classified as up- and downregulated using |logFC| >1 and Benjamini–Hochberg-adjusted *P*-value <0.05 was applied, revealing that the normalization method affects downstream analysis results. Additionally, KEGG pathway enrichment was performed on the DE results of the different normalization methods using g:Profiler (*P*-value < 0.05). Heatmaps displaying Jaccard similarity coefficients for DE gene sets and the enriched KEGG pathways identified by all pairs of normalization methods for the cell culture dataset dB1 (C, E) and for the clinical cancer dataset dB2 (D, F), respectively. The values within the tiles were rounded to one decimal place.

For the sake of simplicity in analyzing dB1, we next focused on the pairwise comparison for the longest experimental time span of the cell culture experiment (D14–D0). Note that all other pairwise comparisons of dB1 are included in the supplement (Supplementary Fig. 12). We calculated the number of up- and downregulated DE proteins using an absolute logFC threshold of 1, and normalization by TMM, EigenMS, MAD, and VSN showed high variability in the number of DE proteins compared to the other methods (Fig. 6A). Additionally, we observed that applying batch effect correction on log2-transformed data (denoted as “log2”) yielded results comparable to those obtained using EigenMS and TMM. EigenMS, TMM, and log2 resulted in >125 DE proteins, whereas <25 DE proteins were detected by applying MAD or VSN. Interestingly, the number of DE proteins identified by normalization with VSN_0.5 but not default VSN was similar to most NMs. The results were further examined by generating volcano plots using PRONE, which revealed that the distribution of *P*-values and logFC values from log2 and TMM are of distinct parabolic shape that are highly pronounced compared to the distribution from the other NMs. Furthermore, the results from log2, TMM, and EigenMS were skewed toward significantly lower logFC values compared to the other methods (Supplementary Fig. 13).

The clinical cancer dataset dB2 revealed higher variations across all NMs (Fig. 6B). However, similar to the cell culture dataset dB1, log2, normalization by TMM and EigenMS resulted in significantly more DE proteins, predominantly upregulated, whereas MAD detected almost no DE proteins. Notably, normalization by NormicsMedian and NormicsVSN showed a higher proportion of downregulated DE proteins compared to other NMs. In the LFQ dataset dB3, normalization by EigenMS and MAD resulted in the highest and lowest number of identified DE proteins, respectively (Supplementary Fig. 14A).

While the amount of identified DE proteins is similar for the majority of NMs for the cell culture dataset dB1 and LFQ dataset dB3, minor variations could potentially influence downstream analyses such as network and gene set enrichment. PRONE introduces a novel feature that assesses the DE protein sets: intersection analyses of the DE results based on Jaccard similarity coefficients. Apart from the methods with high variability in the number of DE proteins in the cell culture dataset dB1 such log2, VSN, TMM, MAD, and EigenMS, the same proteins were identified as DE independent of the NM (Jaccard coefficients >0.9) (Fig. 6C and Supplementary Fig. 15). The exceptions are NormicsMedian and the two NormicsVSN approaches, which exhibited comparatively lower coefficients. The clinical cancer dataset dB2 revealed much lower Jaccard similarity coefficients across all NMs, a contrast not as evident in earlier evaluation stages (Fig. 6D). Also, the LFQ dataset dB3 showed results comparable to the cell culture dataset, with Jaccard coefficients exceeding 0.7 for all NMs except MAD (Supplementary Fig. 14B).

However, we observed that MAD generates notably lower absolute logFC values (Supplementary Fig. 13 and 16A–C), explaining the reduced number of DE proteins identified under the applied logFC threshold for all datasets. To account for this, we applied a reduced logFC threshold of 0.5 to MAD, which not only increased the number of DE proteins but also improved the Jaccard coefficients, indicating greater overlap of DE proteins with those obtained using other NMs (Supplementary Fig. 16D–G).

In line with the DE results, Jaccard similarity coefficients of the enriched KEGG pathways were calculated to assess the overlap of enriched terms across different NMs (see Methods, Fig. 6E and F). The enrichment analysis of the cell culture dataset dB1 indicates that most DE sets of NMs exhibit similar significantly enriched KEGG pathways, suggesting a high degree of functional consistency. In contrast, the clinical cancer dataset shows substantial variability in enriched terms across different NMs. This indicates that, in addition to the identification of distinct DE proteins, their associated biological functions also exhibit substantial variation.

## Discussion

### Evaluation of normalization methods

**Figure 7:**
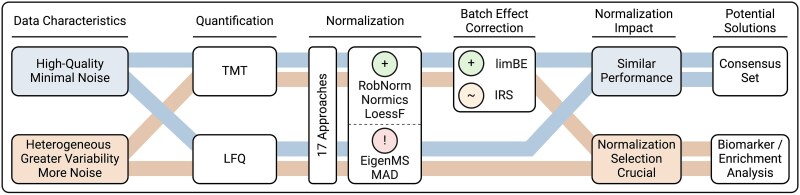
Summary of evaluation study findings. Our evaluation demonstrated that the impact of normalization on the proteomics data analysis is highly dependent on the underlying data characteristics, such as differences between high-quality datasets with minimal noise and heterogeneous datasets exhibiting greater variability. Among the evaluated methods, the normalization methods RobNorm, Normics, and LoessF showed most promising results on the presented spike-in and biological datasets, with limBE being particularly effective in reducing TMT-batch effects present in TMT datasets. Methods were classified as follows: A green plus for most accurate performance, a red exclamation mark for notable limitations, and an orange tilde for methods being less effective than those with a green plus classification.

All NMs, with the exception of TMM, reduced intragroup variation compared to unnormalized data, with EigenMS and MAD being most effective in this regard. MAD was not extensively evaluated before but the findings of EigenMS were confirmed in [2, 9]. Besides MAD and EigenMS, the linear, local regression methods and VSN rank among the most effective techniques for reducing intragroup variation, as demonstrated in [1, 2, 28]. Additionally, with biological data, we demonstrated that batch effect correction is essential for TMT datasets to decrease technical biases (Fig. 7) while maintaining the biological signal, as previously confirmed in [14, 29, 47]. The analysis of the two biological TMT datasets, dB1 and dB2, demonstrated that using the reference samples with IRS for batch effect correction was less effective compared to limBE (Supplementary Figs. 7–11).

Next, we evaluated the impact of NMs on DE analysis. With spike-in data and biological data, we showed that compared to state-of-the-art practice, a reduction of intragroup variation is not directly related to the effectiveness of the NMs according to the DE results. Previous evaluation studies that did not exclusively focus on intragroup variation metrics but also employed DE analysis partially confirmed these findings [2, 9]. Furthermore, we demonstrated that EigenMS-normalized data resulted in a high number of FPs in spike-in data (Fig. 4A and Supplementary Fig. 2). Generally, as already shown in [10], the identification of a high proportion of FPs is observed, which is not recognizable when using AUC values due to the class imbalance. Thus, unlike previous studies [2, 7, 9], we employed F1 score, which uses precision instead of specificity (as in AUC) and thus gives more attention to the problem of FPs in detecting DE proteins.

Despite the challenges in comparing spike-in datasets due to their underlying heterogeneity, resulting from diverse experimental designs and quantification techniques, differences in dataset quality account for the variation in NM performance. TMM exhibited lower F1 scores in dS1–dS2 but performed comparably to the top-performing methods in dS3–dS6. TMM’s trimming process removes proteins with extreme changes; however in dS1–dS2, the high variability of background proteins results in normalization factors being predominantly influenced by background noise and the reduced numbers of retained proteins lead to less robust factors (Table 1 and Supplementary Fig. 1). In contrast, the simple sample shifting methods, including mean and median normalization, suffer from stable background proteins and distinct rates of spike-ins, as observed in dS5–dS6. In these cases, factors are derived from both types of proteins, and the distinct shifts in spike-in proteins cause unintended shifts in background proteins, resulting in increased FPRs (Supplementary Fig. 2A). In this context, more advanced methods, such as RobNorm and LoessF, as well as approaches excluding highly variable proteins from normalization factor estimation, such as Normics and TMM, benefit from a more stable background proteome. The poor performance of cyclic regression methods on dS2 can be attributed to the relatively high proportion of missing values in this dataset (Table 1). Due to differing criteria for handling proteins with missing values (see Supplementary Methods), these methods can introduce additional missing values during normalization, which are subsequently classified as non-DE.

According to the median F1 score calculated over all pairwise comparisons and spike-in datasets, RobNorm is the best-performing NM, closely followed by LoessF which showed no significant difference in performance. Furthermore, NormicsVSN, which has not been previously independently evaluated, ranked among the top-performing methods. In contrast, EigenMS produced the lowest F1 scores, with a comparatively high number of FPs.

Finally, when employing ROTS for DE analysis, FPs decreased, with minimal impact on TPs. Notably, certain methods exhibited a greater benefit from the use of ROTS compared to others, slightly altering the ranking of the NMs. Although LoessF ranked highest with ROTS, its F1 scores were not significantly higher than those resulting from RobNorm, the Normics approaches, and VSN.

Since there is no ground truth for the three biological datasets, we based our evaluation on the number of DE proteins. While data normalized using TMM and EigenMS resulted in a substantially higher number of DE candidates, MAD resulted in a noticeably lower number of DE results. Based on the previous results on spike-in datasets, we hypothesize that the majority of DE proteins stemming from EigenMS may be FPs. The compositional correction achieved through TMM was found to produce comparable results to those obtained without applying explicit normalization before batch effect correction, i.e. on log-transformed data (log2). While data normalized with MAD resulted in relatively low F1 scores in the spike-in datasets, the application of a less stringent logFC threshold in the DE analysis of biological datasets revealed a high overlap in identified DE proteins across other NMs (Supplementary Fig. 15). MAD normalization compresses the variances across samples by centering and scaling the data based on the median absolute deviation, a robust measure of variability that is less sensitive to outliers (Supplementary Fig. 5). This variance compression leads to a decrease in the intragroup variation (Fig. 3 and Supplementary Fig. 11) and systematically lowers logFCs (Supplementary Figs. 4 and 13), which may represent a potential limitation of the method. Hence, the evaluation of the presented spike-in and biological datasets highlighted certain limitations of the EigenMS and MAD approaches, while showing the importance of exploring and assessing emerging methods like RobNorm and the Normics approaches that were specifically developed for application in proteomics research (Fig. 7).

Intersection analyses of the DE proteins and the resulting enriched KEGG pathways in the cell culture dataset dB1 revealed that most NMs consistently produced comparable results. Conversely, the results of the clinical cancer dataset dB2 demonstrated that normalization has a substantial impact on downstream analyses and, hence on the overall conclusions drawn from proteomics data analysis. Hence, based on these two datasets, we hypothesize that for high-quality datasets with minimal noise, most NMs perform similarly, while for more heterogeneous datasets with greater variability and noise, the choice of NM significantly affects the results (Fig. 7). These findings, combined with the F1 scores of the spike-in datasets, indicate that the impact of NMs is highly dataset-specific. For heterogeneous datasets characterized with higher variability, the functional enrichment analysis functionality of PRONE offers a valuable approach for interpreting biological findings and evaluating the effects of NMs. In contrast, for high-quality datasets with minimal noise, such as the cell culture dataset dB1, we hypothesize that utilizing a consensus set of DE proteins could serve as a viable approach for downstream analyses (Fig. 7). However, we emphasize that this hypothesis requires further consideration and validation.

Given the dataset-specific effects and the unknown ground truth in biological datasets, it is advisable to conduct a comparative evaluation of multiple NMs, incorporating both intragroup variation metrics and DE analysis, to guide decisions toward appropriate NMs. However, domain-specific knowledge will be indispensable to account for the dataset specificity depending on the biological questions.

### Study limitations and future work

Despite the comprehensive range of NMs and datasets, this study is subject to potential limitations. First, similar to [48], we want to emphasize that spike-in datasets come with their own limitations. Although spike-in datasets are universally used for evaluating proteomics workflows [1, 2, 7, 8, 10, 17–20, 45, 48–50], they replicate technical variation but lack the biological variation which is inherently present in biological datasets. Furthermore, opposed to biological datasets, in spike-in experiments, the differences between sample groups are driven by a uniform overexpression of all spike-in proteins whose signal is, thus, jointly upregulated [23]. A possible explanation for a high amount of FPs is that spike-in proteins can artificially elevate the apparent intensity of background proteins [23]. Moreover, as spike-in experiments vary drastically in experimental design and the descriptive reporting of spike-in ratios, direct comparison across spike-in datasets is complicated. To the best of our knowledge, no universally accepted approaches currently exist for simulating proteomics data with biological replicates that are suited for the evaluation of proteomics workflows. However, future research would benefit from the development of simulated datasets for inclusion in subsequent evaluations, allowing for a comparative analysis between spike-in datasets and simulated data.

We focused our benchmark primarily on label-free and TMT datasets despite the widespread use of other quantification strategies in the proteomics community, e.g. SILAC and iTRAQ [51, 52]. However, given the minimal data requirements of PRONE, other types of proteomics datasets could be readily incorporated into future evaluations. Additionally, the study was restricted to TMT spike-in datasets measured within a single batch since no suitable publicly available multi-batch spike-in TMT datasets could be found during data collection. Moreover, we focused on protein-centric normalization although proteomic data can be normalized also on peptide levels, as benchmarked in [4, 28].

In this study, we chose to apply POMA for outlier detection prior to normalization, as outliers can substantially influence the normalization process. Nonetheless, a thorough evaluation of the interplay between sample outlier detection and normalization would be of considerable value for future research. Likewise, the systematic evaluation of imputation methods in combination with NMs on DE results provides another reasonable future extension of PRONE.

Previous studies systematically compared different DE methods in proteomics [20, 45, 48, 50], highlighting that methodological choices on DE approaches can significantly impact results. Our comparison of limma and ROTS was not intended as a comprehensive evaluation of DE approaches but rather to emphasize that normalization evaluation outcomes are inherently influenced by the chosen DE method. Future research could expand PRONE’s capabilities by integrating additional DE methods, such as those available in proteiNorm [8] through DAtest [53] integration, and by further exploring their impact on normalization evaluation.

Despite some limitations in this study, the PRONE package already incorporates many key functionalities necessary for further analyses and result evaluation. Importantly, this study represents an important step toward establishing a universal pipeline for proteomics data analysis, with a particular emphasis on normalization.

**Key Points**

- A total of 17 normalization techniques and two batch effect correction methods were systematically assessed in this study across six spike-in and three biological proteomics datasets without known ground truth.
- RobNorm and both variants of Normics, which have not been previously evaluated independently, performed consistently well based on F1 scores across spike-in datasets.
- Differential expression analysis of biological datasets without known ground truth revealed that the choice of normalization has an impact on downstream analyses and the impact is dataset-specific.
- We introduced PRONE, the PRoteomics Normalization Evaluator, an R Bioconductor package coming along with a graphical interface to streamline a proteomics data analysis, especially focusing on data normalization.

## Supplementary Material

Final_Supplement_bbaf201

## Data Availability

The proteomics data of the spike-in and biological datasets without known ground truth utilized in this study and in the vignettes of PRONE are available through Zenodo at 10.5281/zenodo.12657423. The R package and Shiny app source code can be accessed on GitHub at https://github.com/daisybio/PRONE and https://github.com/daisybio/PRONE.Shiny, respectively. Both are distributed under the GPL-3.0 license. Furthermore, for those interested in the source code of the evaluation study, the corresponding R code is available at https://github.com/daisybio/PRONE.Evaluation/.
